# PCR Assay for Rapid Taxonomic Differentiation of Virulent *Staphylococcus aureus* and *Klebsiella pneumoniae* Bacteriophages

**DOI:** 10.3390/ijms24054483

**Published:** 2023-02-24

**Authors:** Maria Kornienko, Dmitry Bespiatykh, Maja Malakhova, Roman Gorodnichev, Nikita Kuptsov, Egor Shitikov

**Affiliations:** Department of Biomedicine and Genomics, Lopukhin Federal Research and Clinical Center of Physical-Chemical Medicine of Federal Medical Biological Agency, 119435 Moscow, Russia

**Keywords:** bacteriophage, phage, bacteriophage typing, therapy, *Staphylococcus aureus*, *Klebsiella pneumoniae*

## Abstract

Phage therapy is now seen as a promising way to overcome the current global crisis in the spread of multidrug-resistant bacteria. However, phages are highly strain-specific, and in most cases one will have to isolate a new phage or search for a phage suitable for a therapeutic application in existing libraries. At an early stage of the isolation process, rapid screening techniques are needed to identify and type potential virulent phages. Here, we propose a simple PCR approach to differentiate between two families of virulent *Staphylococcus* phages (*Herelleviridae* and *Rountreeviridae*) and eleven genera of virulent *Klebsiella* phages (*Przondovirus*, *Taipeivirus*, *Drulisvirus*, *Webervirus*, *Jiaodavirus*, *Sugarlandvirus*, *Slopekvirus*, *Jedunavirus*, *Marfavirus*, *Mydovirus* and *Yonseivirus*). This assay includes a thorough search of a dataset comprising *S. aureus* (*n* = 269) and *K. pneumoniae* (*n* = 480) phage genomes available in the NCBI RefSeq/GenBank database for specific genes that are highly conserved at the taxonomic group level. The selected primers showed high sensitivity and specificity for both isolated DNA and crude phage lysates, which permits circumventing DNA purification protocols. Our approach can be extended and applied to any group of phages, given the large number of available genomes in the databases.

## 1. Introduction

The discovery of antibiotics marked a revolution in healthcare, changing the treatment of infectious diseases for many years to come. However, the emergence and rapid spread of multidrug-resistant (MDR) bacteria has become one of the major threats to public health in the twenty-first century. Based on a comprehensive assessment of the global burden of antimicrobial resistance (AMR), in 2019 AMR was shown to be the third leading cause of death [1]. The usefulness of antibiotics is waning, and it is possible that by the year 2050, MDR infections will be the leading cause of death worldwide [2]. Moreover, the following species pose the greatest danger, among which the proportion of MDR strains exceeds 50%: *Escherichia coli*, *Klebsiella pneumoniae*, *Enterobacter cloacae*, *Staphylococcus* spp., *Enterococcus* spp., *Acinetobacter baumannii*, and *Pseudomonas aeruginosa* [1].

One of the most promising approaches for overcoming the current global crisis of antimicrobial resistance is the usage of (bacterio)phages (viruses that kill bacteria) to treat infections caused by MDR strains. Phage therapy has been shown to be safe and effective against a wide range of bacterial pathogens in both preclinical and clinical studies [3,4,5,6]. However, due to the high specificity of phage–bacteria interactions, in most cases it is necessary to isolate a new phage or to search for a suitable one in existing phage libraries [7].

At the same time, it is important to emphasize that phages are living antimicrobials, which imposes particular requirements on their therapeutic use; specifically, only virulent phages (the reproduction of these bacteriophages always leads to cell lysis; unable to integrate into the genome of an infected bacterium) can be used clinically [8,9]. Whole-genome sequencing (WGS) is gradually introduced to confirm the virulent nature of the phage: knowing the entire phage nucleotide sequence makes it possible to check the genome for the presence of recombination or transposition gene modules (responsible for recombination and/or integration of the phage genome into the host cell genome), toxins, antibiotic resistance genes, and virulence factors [9,10]. Furthermore, the ongoing systematization of information about taxa suitable for therapeutic needs is a result of the active improvement of virus taxonomy, aided by the widespread use of WGS.

A universal hierarchical taxonomic system is being maintained and developed by the International Committee on Taxonomy of Viruses (ICTV). Genomic characteristics are now acknowledged to be the fundamental component of taxonomic classification [11,12]. Several approaches, such as computing genome-wide sequence similarities (ViPTree) [13], network-based genome taxonomy (vConTACT2) [14], and many others, are proposed for viral taxonomic classification. Although WGS-based methods are becoming more and more efficient, they remain rather time-consuming and labor-intensive, especially in the case of emergency selection of individualized phage cocktails. Therefore, the development of a cheaper and faster approach for primary screening is an urgent task for typing existing and de novo isolated phages.

To date, several approaches for phage typing have been proposed. Although alternative methods have been described [15,16], the majority of the studies on phage typing rely on the presence of molecular markers (signature genes) [17,18,19,20]. Furthermore, a significant portion of existing methods is devoted to the typing of temperate bacteriophages [19,20,21,22], which is connected with the need to type bacterial strains. Significantly fewer studies are concerned with the typing of virulent phages; such typing schemes have been proposed only for *Salmonella enterica*, *Escherichia coli* and *Erwinia amylovora* [17].

Here, we propose a novel PCR-based typing scheme for *S. aureus* and *K. pneumoniae* phages. To develop the scheme, a search for taxon-specific orthologous gene families was conducted on a dataset comprising *n* = 749 phage genomes. This assay led to the selection of *n* = 13 primer pairs that could identify two families of virulent *Staphylococcus* phages (*Herelleviridae* and *Rountreeviridae*) and 11 genera of virulent *Klebsiella* phages (*Przondovirus*, *Taipeivirus*, *Drulisvirus*, *Webervirus*, *Jiaodavirus*, *Sugarlandvirus*, *Slopekvirus*, *Jedunavirus*, *Marfavirus*, *Mydovirus* and *Yonseivirus*). The assessment of sensitivity and specificity of bacteriophages and bacteria from laboratory collections showed that the scheme is applicable both for isolated DNA and for crude phage lysates.

## 2. Results

### 2.1. Sample Collection and Phylogenetic Analysis

**Figure 1:**
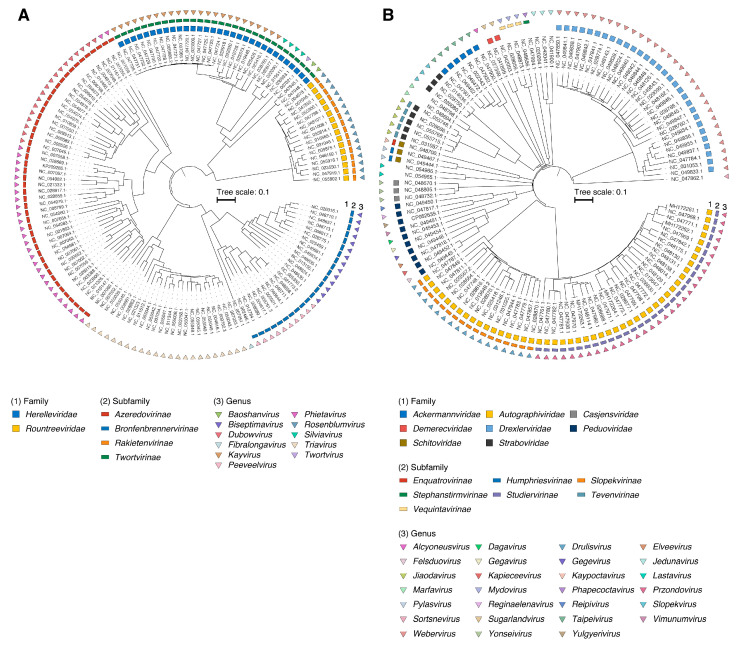
Midpoint-rooted phylogenetic tree of (**A**) *S. aureus* (*n* = 123) and (**B**) *K. pneumoniae* (*n* = 142) phages based on protein distances between genomes. The three external rings indicate the family, subfamily and genus rank, respectively.

To develop a typing scheme, all available *S. aureus* (*n* = 269) and *K. pneumoniae* (*n* = 480) phage genomes in the NCBI RefSeq/GenBank database were used (Table S1A,B). Out of these, ~53% of *Staphylococcus* phages (*n* = 142) and ~25% of *Klebsiella* phages (*n* = 123) have been classified by the ICTV (Figure 1).

Using taxonomy derived from the ICTV database and the vConTACT2 network-based viral classification tool [14], we were able to assign taxonomy to most of the *Staphylococcus* and *Klebsiella* phage genomes retrieved from the NCBI RefSeq/GenBank database (Figures S1 and S2). In total, *Staphylococcus* phages belonged to 2 families, 4 subfamilies, and 11 genera. Meanwhile, a greater diversity of taxonomic groups was found among *Klebsiella* phages: 8 families, 7 subfamilies, and 27 genera (Table 1). For the *n* = 18 *Staphylococcus* and *n* = 178 *Klebsiella* phage genomes, genus-level classification was not assigned.

### 2.2. Identification of Signature Genes Suitable for PCR Typing

We used the following criteria to search for taxon-specific groups of genes suitable for the development of a PCR typing scheme: ≥2 genomes at the genus level, the gene of interest is only present within members of the operational taxonomic unit (OTU), amino acid sequence identity threshold > 60% and average length > 400 bp. From 0 to 207 specific genes were identified for each OTU (Table 1 and Table S2). Phylogenetic trees inferred from protein distances between genomes and trees derived from pangenome analysis based on pairwise distance matrix from gene presence/absence data were found to be largely concordant and showed a similar clustering of OTUs (Figures S3 and S4).

Within the *Staphylococcus* phages, we were unable to identify specific genes that would allow us to accurately differentiate the *Kayvirus, Biseptimavirus, Peeveelvirus* and *Fibralongavirus* genera from the other most-closely related ones. However, at the *Herelleviridae* family level, we found *n* = 8 family-specific genes suitable for typing.

In contrast, for *Klebsiella* phages, we found specific genes for each genus, but not for families. Four genes were found to be orthologous for the *Jiaodavirus* and *Slopekvirus* genera of the *Straboviridae* family, but they were also found in members of the *Marfavirus* genus. Moreover, *n* = 12 genes were found to be common between the *Jiaodavirus* and *Marfavirus* genera, which indicates the close relationship between them.

### 2.3. PCR-Based Typing Scheme for Rapid Phage Classification

Genes were selected for typing if they allowed us to clearly distinguish among genera of virulent phages that have at least five representatives per genus (Table 1). Therefore, primers for *K. pneumoniae* phages were selected for the following genus-specific genes: internal virion protein D (*Przondovirus*), putative tail tube-associated baseplate protein (*Taipeivirus*), capsid protein (*Drulisvirus*), putative major head subunit precursor (*Webervirus*), alpha-glucosyl-transferase (*Jiaodavirus*), putative replicative DNA helicase (*Sugarlandvirus*), putative baseplate hub (*Slopekvirus*), virion structural protein (*Jedunavirus*), hypothetical protein (*Marfavirus*), putative helicase (*Mydovirus*) and DNA polymerase I (*Yonseivirus*). In *Staphylococcus* phages, for the *Rountreeviridae* and *Herelleviridae* families, primers were selected for the gene family encoding the resolvase and major capsid protein (Table 2, all alignments are available via Figshare).

**Figure 2:**
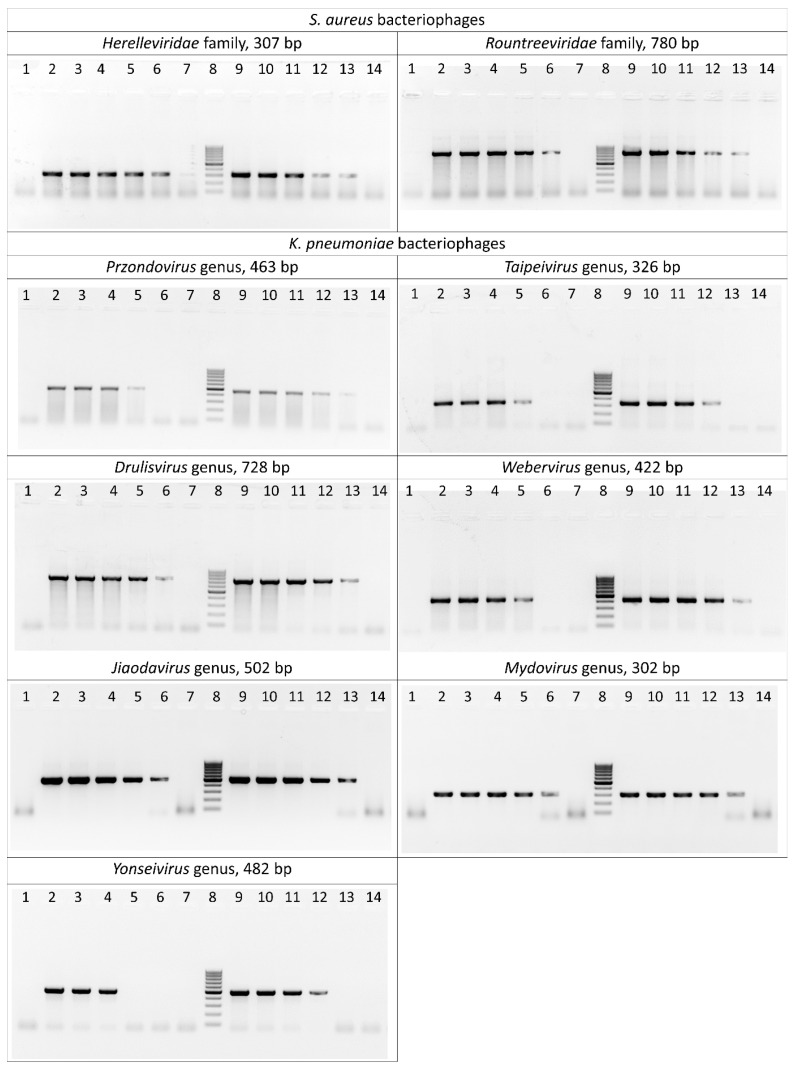
Polymerase chain reaction results for the *S. aureus* and *K. pneumoniae* bacteriophages. Lane 1: negative control; lanes 2–7: serially diluted bacteriophage DNA (15 ng/μL–0.15 pg/μL) as template; lane 8: DNA ladder (the ladder is composed of ten chromatography-purified individual DNA fragments (in base pairs): 1000, 900, 800, 700, 600, 500, 400, 300, 200, 100); lanes 9–14: serially diluted phage lysates (10^9^–10^4^ PFU/mL) as template.

The sensitivity of the selected primer pairs was evaluated both on bacteriophage DNA isolated using phenol–chloroform extraction and directly on crude phage lysates. As a result of serial dilutions of the matrix, it was found that the limit of detection of PCR primers corresponds to a phage DNA concentration ranging from 150 to 1.5 pg/μL. Amplification of lysates was successful at a minimal concentration of 10^4^–10^5^ plaque-forming units per milliliter (PFU/mL) (Figure 2, Table S3).

Specificity of the primers was tested on non-target phage genera from the laboratory collection (*n* = 21) (Table S3). No nonspecific products were detected after the amplification of phages. Additionally, bacterial strains of *S. aureus* (*n* = 21) and *K. pneumoniae* (*n* = 16) were tested These strains were either hosts of the studied phages or were effectively lysed, and were considered as potential hosts. Only several reactions with *K. pneumoniae* strains yielded non-specific results, which appeared as faint bands that were different in size from positive controls. These results were later regarded as negative.

## 3. Discussion

Phages are one of the most abundant and diverse entities of the biosphere; according to some estimates, their number reaches about 10^31^ particles [23]. This wide diversity makes it possible to isolate a specific phage that can lyse almost any of the existing infectious bacterial strains. However, only virulent phages are suitable for phage therapy, and it is not possible to reliably determine the type of phage–bacteria interaction solely on the basis of plaque morphology. Thus, the suitability of a phage for therapy can only be established after acquiring its complete genome via WGS or other additional experiments [24].

In the case of personalized phage therapy, the most important aspect is the issue of time spent on the selection of a suitable phage, and therefore a pre-WGS screening that would be able to exclude temperate phages from the analysis would be greatly beneficial. It has been shown that phages belonging to the same taxonomic unit (genus, family) are characterized by the same type of interaction with the cell (virulent or temperate) [25], which makes it possible to take the taxonomic affiliation of phages as a basis for a rapid preliminary screening.

In addition to characterizing the type of phage-bacteria interaction, taxonomic affiliation makes it possible to determine the approximate size of the phage genome [26,27], which is also important for WGS analysis. Since the size of phage genomes varies greatly, this must be taken into account at the DNA library preparation step in order to achieve optimal coverage and high sequencing quality [27].

To create a PCR typing system, we limited ourselves to *S. aureus* and *K. pneumoniae* phages. Both bacterial species cause a wide range of infectious diseases and belong to the so-called ESKAPE (*Enterococcus faecium*, *S. aureus*, *K. pneumoniae*, *Acinetobacter baumannii*, *Pseudomonas aeruginosa*, and *Enterobacter* species) group of pathogens, characterized by the high resistance of clinical strains to antimicrobial agents, which makes them important potential targets for phage therapy [1,4]. On the other hand, phages of these species are often well-characterized and a sufficient number of cases of their successful application have been described [4].

*Staphylococcus* phages are grouped into three major families: *Herelleviridae* (former *Myoviridae*), *Rountreeviridae* (former *Podoviridae*), and unclassified family (former *Siphoviridae*). The *Klebsiella* phages are classified into *Ackermannviridae* (former *Myoviridae*), *Autographiviridae* (former *Podoviridae*), *Casjensviridae* (former *Siphoviridae*), *Demerecviridae* (former *Siphoviridae*), *Drexlerviridae* (former *Siphoviridae*), *Peduoviridae* (former *Myoviridae*), *Schitoviridae* (former *Podoviridae*) and *Straboviridae* (former *Myoviridae*) families, as well as unclassified genera (formerly belonging to the families *Myoviridae* and *Podoviridae*) (Figure 1, Table 1). All known virulent *S. aureus* phages belong to the *Herelleviridae* and *Rountreeviridae* families [28], while temperate bacteriophages are not found in these families. Among the *K. pneumoniae* phages, *n* = 20 genera were found, comprising exclusively virulent phages. The aforementioned corroborates that the type of life cycle closely correlates with the taxonomic affiliation of phages.

Thirteen primer pairs were selected based on the gene alignments of the virulent *S. aureus* and *K. pneumoniae* phages (Table 2). In a number of cases, the genes chosen in this study, such as major capsid protein and DNA polymerase, match those previously proposed for phage typing and classification [17,29,30]. These genes play a key role in the physiology of phages and are widely distributed among various families. As previously established, their use as target genes for PCR typing schemes allows viruses to be reliably differentiated irrespective of the mosaic nature of their genome, presumably due to limited horizontal gene transfer [31]. This study made it possible to significantly expand the list of conservative genes specific to certain taxonomic units, which can be used in further phylogenetic studies (Table S2). For example, we have found eight specific genes for the family *Herelleviridae*, which are part of the previously described core genes of this family [32].

In silico, the selected primers showed the ability to detect all currently known *S. aureus* phages (*Herelleviridae* and *Rountreeviridae* families) associated with a virulent lifestyle. In the case of *K. pneumoniae* phages, the proposed PCR scheme is able to differentiate *n* = 11 from *n* = 20 genera of virulent bacteriophages. The vast majority of the studied virulent *K. pneumoniae* phages (92.4%) belong to phages from the aforementioned eleven genera.

Testing of the thirteen PCR schemes was carried out on a laboratory collection of phages, the genomes of which were not used for the primer design. Results obtained confirmed that the selected loci are conservative, and the primers have good specificity. Moreover, the limit of detection of the scheme was found to be rather high, which allows it to be used both when working with isolated DNA and with lysates when searching for a specific phage.

The proposed PCR assay has a number of limitations that must be taken into account. Although *Sugarlandvirus*, *Slopekvirus*, *Jedunavirus*, and *Marfavirus* primers showed high specificity in silico, they were not tested in vitro due to the absence of these phages in our laboratory collection. Moreover, for some primer systems, an experimental confirmation of sensitivity and specificity was carried out on a single phage, so sensitivity limits require further confirmation. Finally, it is necessary to consider the fact that several samples of *K. pneumoniae* showed nonspecific products, but we believe this issue can be overcome by introducing an additional control in the form of phage-free bacterial genomic DNA.

## 4. Materials and Methods

### 4.1. Genome Sampling and Annotation

A dataset comprising *n* = 749 complete phage genomes was retrieved from the NCBI RefSeq/GenBank database using ncbi-acc-download v.0.2.8 (https://github.com/kblin/ncbi-acc-download; accessed on 7 June 2022). Gene prediction and annotation were carried out using Prokka v.1.14.6 [33], supplemented with the pVOG [34], PHROG v.3 [35] and Caudovirales (http://s3.climb.ac.uk/ADM_share/Caudovirales.tar.gz; accessed on 7 June 2022) databases.

### 4.2. Phylogenetic Analysis

Trees based on pairwise distances between phage genomes were inferred using a standalone version of ViPTree v.1.1.2 [13]. For 265 genomes, the official taxonomy was derived from the ICTV Master Species List 2021.v1 (https://talk.ictvonline.org/files/master-species-lists/m/msl/13425/download; accessed on 7 June 2022). Phage genomes that were not present in the ICTV database were classified using vConTACT2 v.0.9.22 with default parameters using the “ProkaryoticViralRefSeq211-Merged” database [14] to obtain a taxonomic affiliation at the family or genus rank. Furthermore, a phage’s lifestyle (virulent or temperate) was predicted from conserved protein domains using BACPHLIP v.0.9.6 [10]. All trees were midpoint-rooted and visualized in RStudio v.2022.02.3 with R v.4.1.2 using ggtree v.3.2.1 [36], ggtreeExtra v.1.4.2 [37], ggplot2 v.3.3.5 [38], ggnewscale v.0.4.7 (https://zenodo.org/record/6385112; accessed on 7 June 2022), ggsci v.2.9 (https://github.com/nanxstats/ggsci; accessed on 7 June 2022), ggstar v.1.0.3 (https://github.com/xiangpin/ggstar; accessed on 7 June 2022), qualpalr v.0.4.3 [39] and here v.1.0.1 [40].

### 4.3. Pangenome Analysis and Homologues Identification

The resulting annotations in GFF3 format derived using Prokka were used for pangenome construction. Pangenome analyses were carried out using PIRATE v.1.0.4 [41] with amino acid identity thresholds of 30, 40, 50, 60, 70, 80, 90 and 95. SeqKit v.2.2.0 [42] and Seqtk v.1.3-r106 (https://github.com/lh3/seqtk; accessed on 7 June 2022) were used for handling sequences in FASTA format.

### 4.4. Primer Design

Genes were selected for potential use in the typing system for each operational taxonomic unit Orthologous genes with an identity of >60% and average length of >400 bp were used for further analysis. Orthologous gene family alignment was performed for each pair of genes using MAFFT v.7.490 [43] under default parameters. Primers were designed using BioEdit v.7.0.5.3 (http://www.mbio.ncsu.edu/BioEdit/bioedit.html; accessed on 7 June 2022) and OLIGO v.6.31 (Molecular Biology Insights Inc., Cascade, CO, USA). The NCBI Primer-BLAST online tool (https://www.ncbi.nlm.nih.gov/tools/primer-blast/; accessed on 7 June 2022) was used to assess primer pair specificity [44]. The primers used in this study and PCR product lengths are listed in Table 1.

### 4.5. PCR Verification

Validation of the PCR typing assays was carried out on a laboratory collection of bacteriophages, including *S. aureus* (*n* = 6) bacteriophages from the families *Herelleviridae* and *Rountreeviridae* and *K. pneumoniae* (*n* = 15) bacteriophages from seven different genera (*Przondovirus*, *Taipeivirus*, *Drulisvirus*, *Webervirus*, *Jiaodavirus*, *Mydovirus* and *Yonseivirus*) (Table S3). A standard phenol–chloroform extraction protocol was used for phage DNA isolation [45]. DNA concentration was determined using a Qubit 2.0 spectrophotometer (Life Technologies, Darmstadt, Germany).

The standard PCR was carried out in 25 µL of the reaction mixture (66 mM Tris–HCl (pH 9.0), 16.6 mM (NH_4_)_2_SO_4_, 2.5 mM MgCl_2_, 250 µM of each dNTP, 1 U of Taq DNA polymerase (Promega, Madison, WI, USA) and 5 pmol of each primer). About 10 ng of phage DNA was used as a template for PCR. PCR was performed in a DNA Engine Tetrad (MJ Research, Inc., Saint-Bruno-de-Montarville, Quebec, Canada) at 96 °C for 2 min, followed by 35 cycles of 30 s at 95 °C, 20 s at 56 °C for *Staphylococcus* phage primers and 60 °C for *Klebsiella* phage primers, and 40 s at 72 °C. Amplification products were analyzed by 2% agarose gel electrophoresis followed by ethidium bromide staining.

The limit of detection of the PCR system was determined by serial dilutions of DNA template (15 ng/μL–0.15 pg/μL). In addition, crude phage lysates without preliminary purification, obtained by growing bacteriophages on their host strains in lysogeny broth (LB) medium, were used as a matrix. For PCR, 5 μL of lysates containing from 10^9^ to 10^4^ PFU/mL were taken.

PCR specificity was evaluated by cross-testing the selected primers on bacteriophages from other genera (Table S3). DNA from bacterial host strains and strains from the laboratory collection (*S. aureus* (*n* = 21) belonging to 21 multi-locus sequencing typing (MLST) sequence types (STs) and *K. pneumoniae* (*n* = 16) belonging to 16 MLST STs) were also used for testing. MLST was performed based on standard schemes [https://pubmlst.org/; accessed on 7 June 2022]. The primers for the *arcC* and *rpoB* housekeeping genes from *S. aureus* and *K. pneumoniae*, respectively, were used as positive controls.

## 5. Conclusions

The introduction of phage therapy in clinical practice has led to the development of criteria that a potential phage preparation must meet. The initial stage in the creation of such preparations, however, is currently rather empirical in nature, with phages that effectively lyse a certain pathogenic strain of a certain bacterial species being selected during screening.

In this work, we used generalized genomic data to create a PCR typing scheme for *S. aureus* and *K. pneumoniae* phages. This scheme could be used for a preliminary rapid screening of phages. The approach described here can be applied to other bacterial species, which will ultimately facilitate the selection of phages for further study and their application for therapeutic purposes.

## Figures and Tables

**Table 1:** Taxonomy of *S. aureus* and *K. pneumoniae* bacteriophages.

Family	Subfamily	Genus	NCBI (incl. ICTV)	Average Genome Length, Kbp	Orthologous Gene Families (N)
***Staphylococcus aureus*** **phages**					
*Herelleviridae*	*Twortvirinae*	Baoshanvirus *	2 (2)	146.1	14
		Kayvirus *	54 (24)	142.0	0
		Silviavirus *	9 (4)	135.9	3
		Twortvirus *	1 (1)	130.7	
		Unclassified *	5		
	Unclassified	Unclassified *	1		
*Rountreeviridae*	*Rakietenvirinae*	Rosenblumvirus *	29 (16)	17.7	9
	*Azeredovirinae*	*Dubowvirus*	32 (18)	43.1	4
		*Phietavirus*	39 (29)	44.4	2
	*Bronfenbrennervirinae*	*Biseptimavirus*	20 (14)	42.6	0
		*Peeveelvirus*	22 (10)	42.0	0
		*Fibralongavirus*	1 (1)	41.3	0
		*Triavirus*	42 (23)	45.8	3
Unclassified	Unclassified	Unclassified	12		
***Klebsiella pneumoniae*** **phages**					
*Ackermannviridae*		Taipeivirus *	7 (6)	158.5	63
*Autographiviridae*	*Slopekvirinae*	Drulisvirus *	48 (17)	44.1	13
	*Studiervirinae*	Przondovirus *	79 (31)	40.6	10
		Unclassified *	12		
	Unclassified	Unclassified	4		
*Casjensviridae*		Yonseivirus *	9 (3)	58.8	2
*Demerecviridae*		Sugarlandvirus *	11 (2)	112.6	55
*Drexlerviridae*		Webervirus *	75 (30)	49.6	7
*Peduoviridae*		*Reipivirus*	1 (1)	38.3	
		*Dagavirus*	1 (1)	39.0	
		*Elveevirus*	1 (1)	33.5	
		Felsduovirus *	1 (1)	18.2	
		*Gegavirus*	1 (1)	33.8	
		*Gegevirus*	1 (1)	39.6	
		Kapieceevirus *	1 (1)	32.3	
		*Reginaelenavirus*	1 (1)	35.1	
		Vimunumvirus *	1 (1)	34.1	
		*Yulgyerivirus*	1 (1)	32.3	
	Unclassified	Unclassified	61		
*Schitoviridae*	*Enquatrovirinae*	Kaypoctavirus *	1 (1)	73.7	
	*Humphriesvirinae*	Pylasvirus *	2 (2)	70.5	12
*Straboviridae*	*Tevenvirinae*	Jiaodavirus *	15 (5)	116.8	23
		Slopekvirus *	9 (3)	175.4	39
	*Vequintavirinae*	Mydovirus *	6 (3)	143.7	41
	*Stephanstirmvirinae*	Phapecoctavirus *	1 (1)	150.9	
		Alcyoneusvirus *	3 (1)	347.0	207
		Jedunavirus *	12 (3)	47.5	12
		*Lastavirus*	3 (2)	62.1	18
		Marfavirus *	10 (2)	170.1	16
		Sortsnevirus *	1 (1)	42.5	
Unclassified	Unclassified	Unclassified	101		

* Members of this genus are virulent bacteriophages.

**Table 2:** Primer sets for taxon differentiation.

Target	Taxon	Primer Name	Nucleotide Sequence (5′—3′)	Product Size (bp)
***S. aureus*** **bacteriophage typing**				
resolvase	*Herelleviridae* family	Herell_f	GAATTAACTTCTTGGTGGGG	307
		Herell_r	ATACTTTTTCATCATAMGGTAA	
major capsid protein	*Rountreeviridae* family	Rountr_f	TCAATTTCCAAACATTAGCAG	780
		Rountr_f	GGATTTACATCTTGGTCAGTA	
***K. pneumoniae*** **bacteriophage typing**				
internal virion protein D	*Przondovirus* genus	Przond_f	CGTACAACCAAGGKGAAGG	463
		Przond_r	TCCGTGAACACATCRTACCC	
putative tail tube associated baseplate protein	*Taipeivirus* genus	Taipei_f	AGTTCTGAACACCAAAGGC	326
		Taipei_r	CCAACTCAGAGCCGTTCC	
capsid protein	*Drulisvirus* genus	Drulis_f	CGCTCCGTAACGATAAGATG	728
		Drulis_r	ACGCAGACCGATGTTGTAC	
putative major head subunit precursor	*Webervirus* genus	Weber_f	CCTATGATGACGACTCAAAC	422
		Weber_r	ATTGCCAGCCATCTTATCAG	
alpha-glucosyl-transferase	*Jiaodavirus* genus	Jiaoda_f	TGAACATCAAAGCAATTCGTG	502
		Jiaoda_r	AACCACAGAATGCCAGAATC	
putative replicative DNA helicase	*Sugarlandvirus* genus	Sugarl_f	GATCTACCAAGCTGTCCAG	307
		Sugarl_r	AGTCGTTGTTACTCGTTCC	
putative baseplate hub	*Slopekvirus* genus	Slopek_f	TCAAAGAACAATACGAAGAGG	411
		Slopek_r	TTGCCATTGCTTCCAGAGAG	
virion structural protein	*Jedunavirus* genus	Jeduna_f	ACTTCTATTGTCATGGCTGG	349
		Jeduna_r	CACCTTACAGTTTAGCGTC	
hypothetical protein	*Marfavirus* genus	Marfa_f	GCACCTGAAGGCATTACCC	252
		Marfa_r	CCCATCAATAGAATAAAGCAC	
putative helicase	*Mydovirus* genus	Mydo_f	GATCGAAAAGAATGTCTGGG	ast302
		Mydo_r	TTGGTCTACGATAATATCACG	
DNA polymerase I	*Yonseivirus* genus	Yonsei_f	GCACGCCGACCTATCCCG	482
		Yonsei_r	GCCACGGTCATTGATAAGC	

Primer pairs were tested on the phages from a laboratory collection comprising *Staphylococcus* phages *Herelleviridae* (*n* = 3) and *Rountreeviridae* (*n* = 3) and *K. pneumoniae* phages *Przondovirus* (*n* = 2), *Taipeivirus* (*n* = 1), *Drulisvirus* (*n* = 6), *Webervirus* (*n* = 1), *Jiaodavirus* (*n* = 1) and *Mydovirus* (*n* = 3). In all cases, expected amplicon sizes were experimentally confirmed (Figure 2, Table S3).

## Data Availability

Annotated genomes, sequence alignments, phylogenetic trees, and supplementary data are available at FigShare (https://doi.org/10.6084/m9.figshare.20337603.v1, accessed on 7 June 2022). The custom code underlying the analysis and scripts for generating the figures are available at GitHub (https://github.com/dbespiatykh/Kornienko_et_al_2022_phagePCRtyping.git, accessed on 7 June 2022).

## Supplementary Materials

The following supporting information can be downloaded at: https://www.mdpi.com/article/10.3390/ijms24054483/s1.

## References

[1] Murray C.J., Ikuta K.S., Sharara F., Swetschinski L., Robles Aguilar G., Gray A., Han C., Bisignano C., Rao P., Wool E. (2022). Global burden of bacterial antimicrobial resistance in 2019: A systematic analysis. Lancet.

[2] (2014). Review on Antimicrobial Resistance. Antimicrobial Resistance: Tackling a Crisis for the Health and Wealth of Nations.

[3] Leitner L., Ujmajuridze A., Chanishvili N., Goderdzishvili M., Chkonia I., Rigvava S., Chkhotua A., Changashvili G., McCallin S., Schneider M.P. (2021). Intravesical bacteriophages for treating urinary tract infections in patients undergoing transurethral resection of the prostate: A randomised, placebo-controlled, double-blind clinical trial. Lancet Infect. Dis..

[4] Suh G.A., Lodise T.P., Tamma P.D., Knisely J.M., Alexander J., Aslam S., Barton K.D., Bizzell E., Totten K.M.C., Campbell J.L. (2022). Considerations for the Use of Phage Therapy in Clinical Practice. Antimicrob. Agents Chemother..

[5] Petrovic Fabijan A., Lin R.C.Y., Ho J., Maddocks S., Ben Zakour N.L., Iredell J.R., Khalid A., Venturini C., Chard R., Morales S. (2020). Safety of bacteriophage therapy in severe Staphylococcus aureus infection. Nat. Microbiol..

[6] Dedrick R.M., Guerrero-Bustamante C.A., Garlena R.A., Russell D.A., Ford K., Harris K., Gilmour K.C., Soothill J., Jacobs-Sera D., Schooley R.T. (2019). Engineered bacteriophages for treatment of a patient with a disseminated drug-resistant Mycobacterium abscessus. Nat. Med..

[7] Loh B., Leptihn S. (2020). A Call For a Multidisciplinary Future of Phage Therapy to Combat Multi-drug Resistant Bacterial Infections. Infect. Microbes Dis..

[8] Cui Z., Guo X., Feng T., Li L. (2019). Exploring the whole standard operating procedure for phage therapy in clinical practice. J. Transl. Med..

[9] Harper D.R. (2018). Criteria for Selecting Suitable Infectious Diseases for Phage Therapy. Viruses.

[10] Hockenberry A.J., Wilke C.O. (2021). BACPHLIP: Predicting bacteriophage lifestyle from conserved protein domains. PeerJ.

[11] Turner D., Kropinski A.M., Adriaenssens E.M. (2021). A Roadmap for Genome-Based Phage Taxonomy. Viruses.

[12] Dutilh B.E., Varsani A., Tong Y., Simmonds P., Sabanadzovic S., Rubino L., Roux S., Munoz A.R., Lood C., Lefkowitz E.J. (2021). Perspective on taxonomic classification of uncultivated viruses. Curr. Opin. Virol..

[13] Nishimura Y., Yoshida T., Kuronishi M., Uehara H., Ogata H., Goto S. (2017). ViPTree: The viral proteomic tree server. Bioinformatics.

[14] Bin Jang H., Bolduc B., Zablocki O., Kuhn J.H., Roux S., Adriaenssens E.M., Brister J.R., Kropinski A.M., Krupovic M., Lavigne R. (2019). Taxonomic assignment of uncultivated prokaryotic virus genomes is enabled by gene-sharing networks. Nat. Biotechnol..

[15] Grunwald A., Dahan M., Giesbertz A., Nilsson A., Nyberg L.K., Weinhold E., Ambjornsson T., Westerlund F., Ebenstein Y. (2015). Bacteriophage strain typing by rapid single molecule analysis. Nucleic Acids Res..

[16] Stverakova D., Sedo O., Benesik M., Zdrahal Z., Doskar J., Pantucek R. (2018). Rapid Identification of Intact Staphylococcal Bacteriophages Using Matrix-Assisted Laser Desorption Ionization-Time-of-Flight Mass Spectrometry. Viruses.

[17] Born Y., Knecht L.E., Eigenmann M., Bolliger M., Klumpp J., Fieseler L. (2019). A major-capsid-protein-based multiplex PCR assay for rapid identification of selected virulent bacteriophage types. Arch. Virol..

[18] Moisan M., Moineau S. (2012). Multilocus sequence typing scheme for the characterization of 936- like phages infecting Lactococcus lactis. Appl. Environ. Microbiol..

[19] Doria F., Napoli C., Costantini A., Berta G., Saiz J.C., Garcia-Moruno E. (2013). Development of a new method for detection and identification of Oenococcus oeni bacteriophages based on endolysin gene sequence and randomly amplified polymorphic DNA. Appl. Environ. Microbiol..

[20] Kahankova J., Pantucek R., Goerke C., Ruzickova V., Holochova P., Doskar J. (2010). Multilocus PCR typing strategy for differentiation of *Staphylococcus aureus* siphoviruses reflecting their modular genome structure. Environ. Microbiol..

[21] Ko D.S., Seong W.J., Kim D., Kim E.K., Kim N.H., Lee C.Y., Kim J.H., Kwon H.J. (2018). Molecular prophage typing of *Staphylococcus aureus* isolates from bovine mastitis. J. Vet. Sci..

[22] Sanchini A., Del Grosso M., Villa L., Ammendolia M.G., Superti F., Monaco M., Pantosti A. (2014). Typing of Panton-Valentine leukocidin-encoding phages carried by methicillin-susceptible and methicillin-resistant Staphylococcus aureus from Italy. Clin. Microbiol. Infect..

[23] Hendrix R.W., Smith M.C.M., Burns R.N., Ford M.E., Hatfull G.F. (1999). Evolutionary relationships among diverse bacteriophages and prophages: All the world’s a phage. Proc. Natl. Acad. Sci. USA.

[24] Keen E.C., Adhya S.L., Wormser G.P. (2015). Phage Therapy: Current Research and Applications. Clin. Infect. Dis..

[25] Grose J.H., Casjens S.R. (2014). Understanding the enormous diversity of bacteriophages: The tailed phages that infect the bacterial family Enterobacteriaceae. Virology.

[26] Philipson C.W., Voegtly L.J., Lueder M.R., Long K.A., Rice G.K., Frey K.G., Biswas B., Cer R.Z., Hamilton T., Bishop-Lilly K.A. (2018). Characterizing Phage Genomes for Therapeutic Applications. Viruses.

[27] Russell D.A. (2018). Sequencing, Assembling, and Finishing Complete Bacteriophage Genomes. Methods Mol. Biol..

[28] Deghorain M., Van Melderen L. (2012). The staphylococci phages family: An overview. Viruses.

[29] Henry M., Bobay L.-M., Chevallereau A., Saussereau E., Ceyssens P.-J., Debarbieux L. (2015). The Search for Therapeutic Bacteriophages Uncovers One New Subfamily and Two New Genera of Pseudomonas-Infecting Myoviridae. PLoS ONE.

[30] Adriaenssens E.M., Ackermann H.-W., Anany H., Blasdel B., Connerton I.F., Goulding D., Griffiths M.W., Hooton S.P., Kutter E.M., Kropinski A.M. (2012). A suggested new bacteriophage genus: “Viunalikevirus”. Arch. Virol..

[31] Smith K.C., Castro-Nallar E., Fisher J.N., Breakwell D.P., Grose J.H., Burnett S.H. (2013). Phage cluster relationships identified through single gene analysis. BMC Genom..

[32] Barylski J., Enault F., Dutilh B.E., Schuller M.B.P., Edwards R.A., Gillis A., Klumpp J., Knezevic P., Krupovic M., Kuhn J.H. (2020). Analysis of Spounaviruses as a Case Study for the Overdue Reclassification of Tailed Phages. Syst. Biol..

[33] Seemann T. (2014). Prokka: Rapid prokaryotic genome annotation. Bioinformatics.

[34] Grazziotin A.L., Koonin E.V., Kristensen D.M. (2017). Prokaryotic Virus Orthologous Groups (pVOGs): A resource for comparative genomics and protein family annotation. Nucleic Acids Res..

[35] Terzian P., Olo Ndela E., Galiez C., Lossouarn J., Perez Bucio R.E., Mom R., Toussaint A., Petit M.A., Enault F. (2021). PHROG: Families of prokaryotic virus proteins clustered using remote homology. NAR Genomics Bioinforma..

[36] Yu G. (2020). Using ggtree to Visualize Data on Tree-Like Structures. Curr. Protoc. Bioinforma..

[37] Xu S., Dai Z., Guo P., Fu X., Liu S., Zhou L., Tang W., Feng T., Chen M., Zhan L. (2021). ggtreeExtra: Compact Visualization of Richly Annotated Phylogenetic Data. Mol. Biol. Evol..

[38] Wickham H. (2016). Ggplot2: Elegant Graphics for Data Analysis.

[39] Larsson J. Qualpalr: Automatic Generation of Qualitative Color Palettes. R Package Version 0.4.3. https://CRAN.R-project.org/package=qualpalr.

[40] Muller K. Here: A Simpler Way to Find Your Files. R Package Version 1.0.1. https://CRAN.R-project.org/package=here.

[41] Bayliss S.C., Thorpe H.A., Coyle N.M., Sheppard S.K., Feil E.J. (2019). PIRATE: A fast and scalable pangenomics toolbox for clustering diverged orthologues in bacteria. Gigascience.

[42] Shen W., Le S., Li Y., Hu F. (2016). SeqKit: A Cross-Platform and Ultrafast Toolkit for FASTA/Q File Manipulation. PLoS ONE.

[43] Katoh K., Standley D.M. (2013). MAFFT Multiple Sequence Alignment Software Version 7: Improvements in Performance and Usability. Mol. Biol. Evol..

[44] Ye J., Coulouris G., Zaretskaya I., Cutcutache I., Rozen S., Madden T.L. (2012). Primer-BLAST: A tool to design target-specific primers for polymerase chain reaction. BMC Bioinform..

[45] Sambrook J., Fritsch E.F., Maniatis T. (2012). Molecular Cloning: A Laboratory Manual 2012 Edition (Final).

