# Supplementary material for: PCR Assay for Rapid Taxonomic Differentiation of Virulent Staphylococcus aureus and Klebsiella pneumoniae Bacteriophages
Source: Int J Mol Sci. 2023 Feb 24;24(5):4483. doi: 10.3390/ijms24054483 (PMC10003202; doi:10.3390/ijms24054483)
Supplement: Supplementary file 1 [file ijms-24-04483-s001.zip › supplementary_figures.pdf]

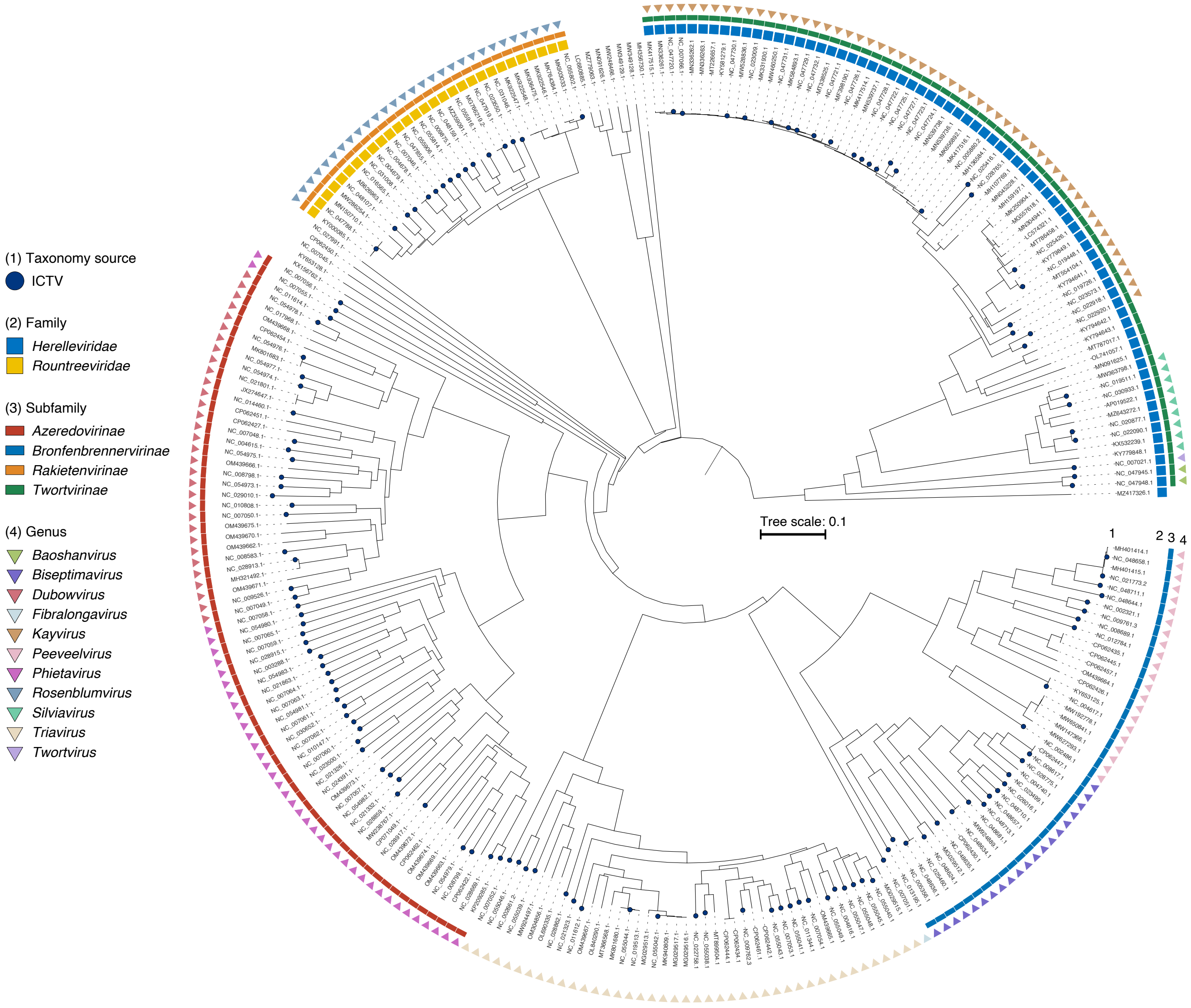


## Supplementary Figure S1

Midpoint-rooted phylogenetic tree of $n$ = 269 *S. aureus* phages based on protein distances between genomes.

The three external rings indicate the family, subfamily and genus rank, respectively. Genomes with available ICTV taxonomy are shown as dots on tip ends.

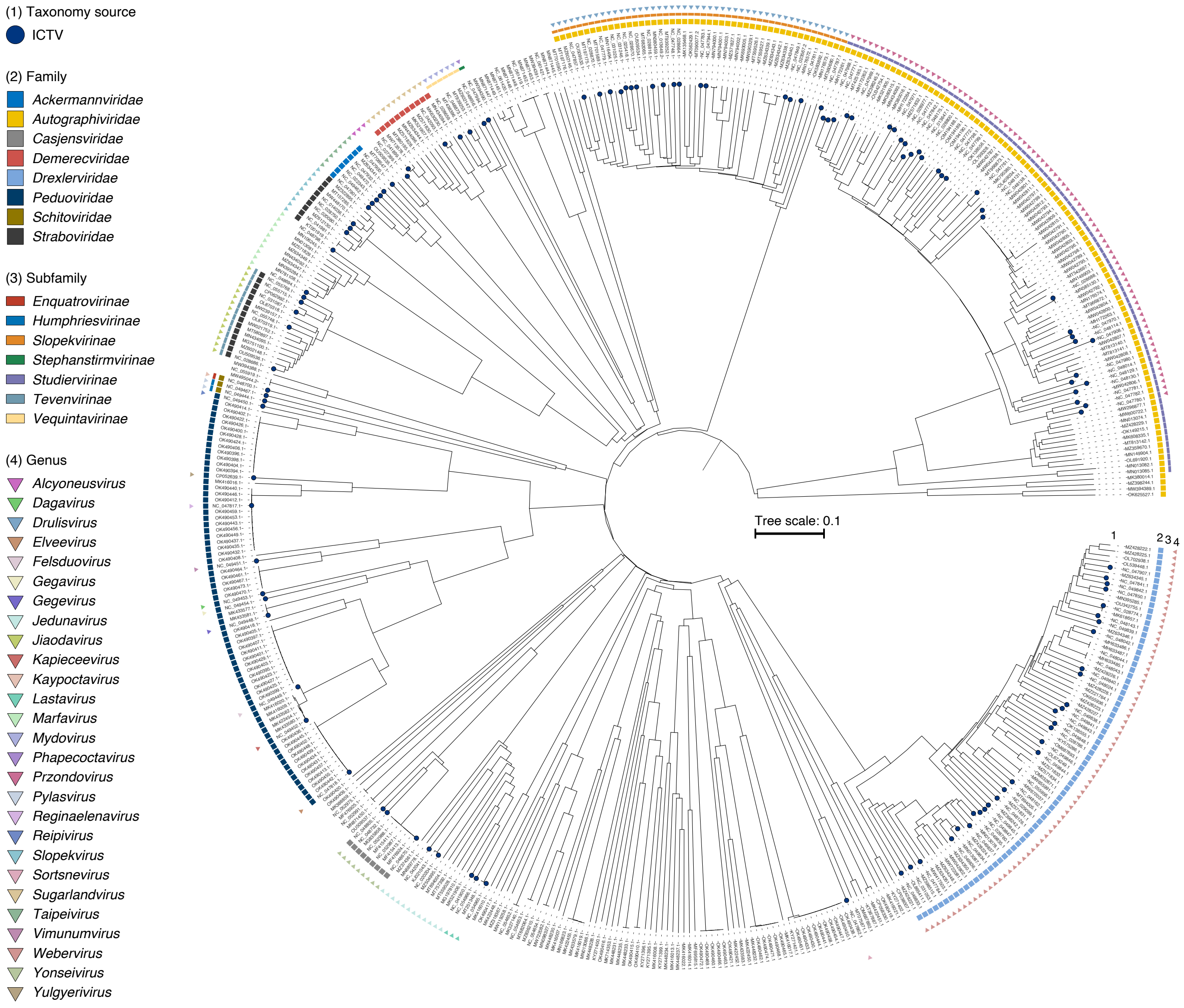


## Supplementary Figure S2

Midpoint-rooted phylogenetic tree of $n$ = 480 *K. pneumoniae* phages based on protein distances between genomes.

The three external rings indicate the family, subfamily and genus rank, respectively. Genomes with available ICTV taxonomy are shown as dots on tip ends.

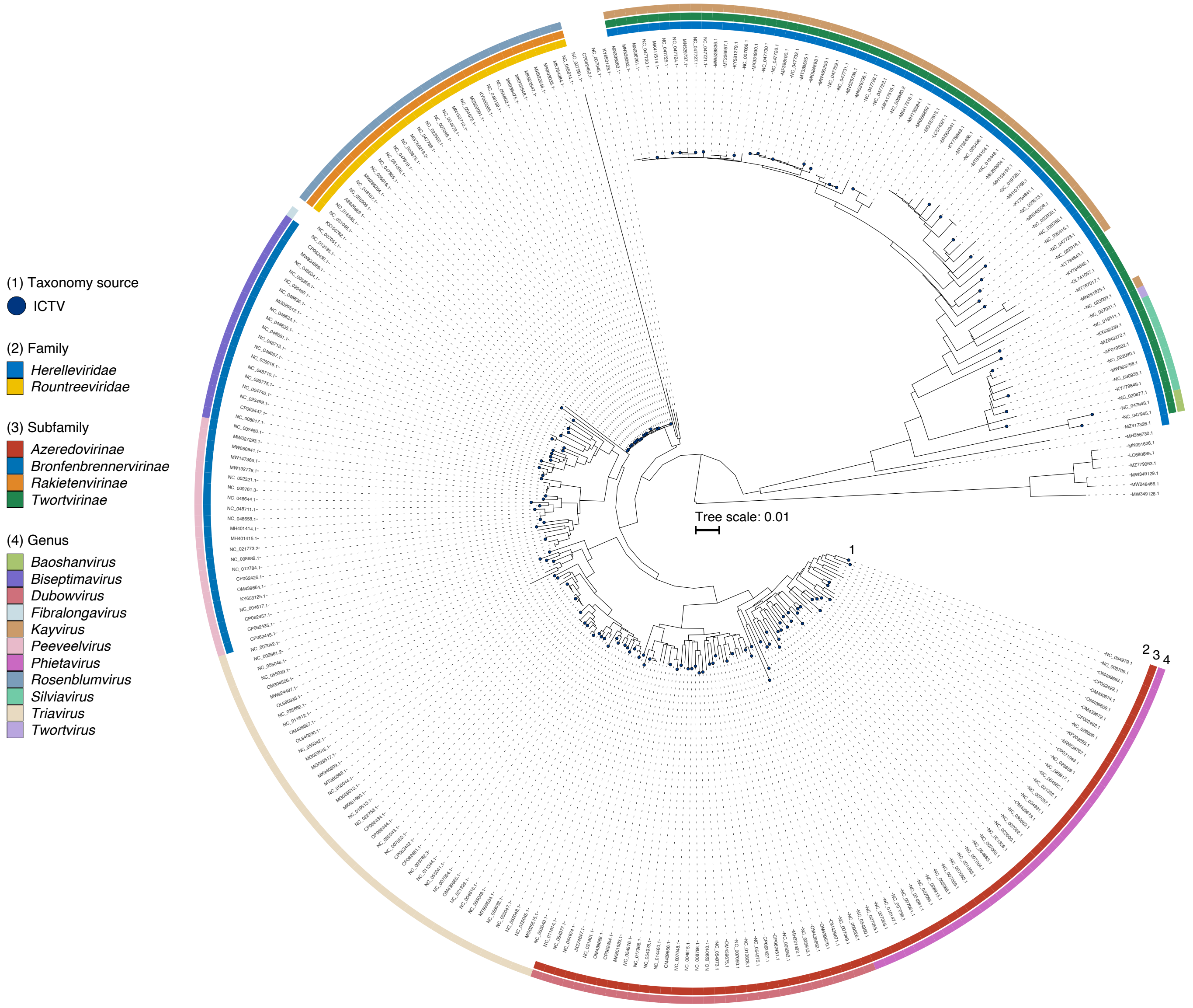


**Supplementary Figure S3**

Midpoint-rooted phylogenetic tree of $n$ = 269 *S. aureus* phages based on pairwise distance matrix from gene presence/absence data.

The three external rings indicate the family, subfamily and genus rank, respectively. Genomes with available ICTV taxonomy are shown as dots on tip ends.

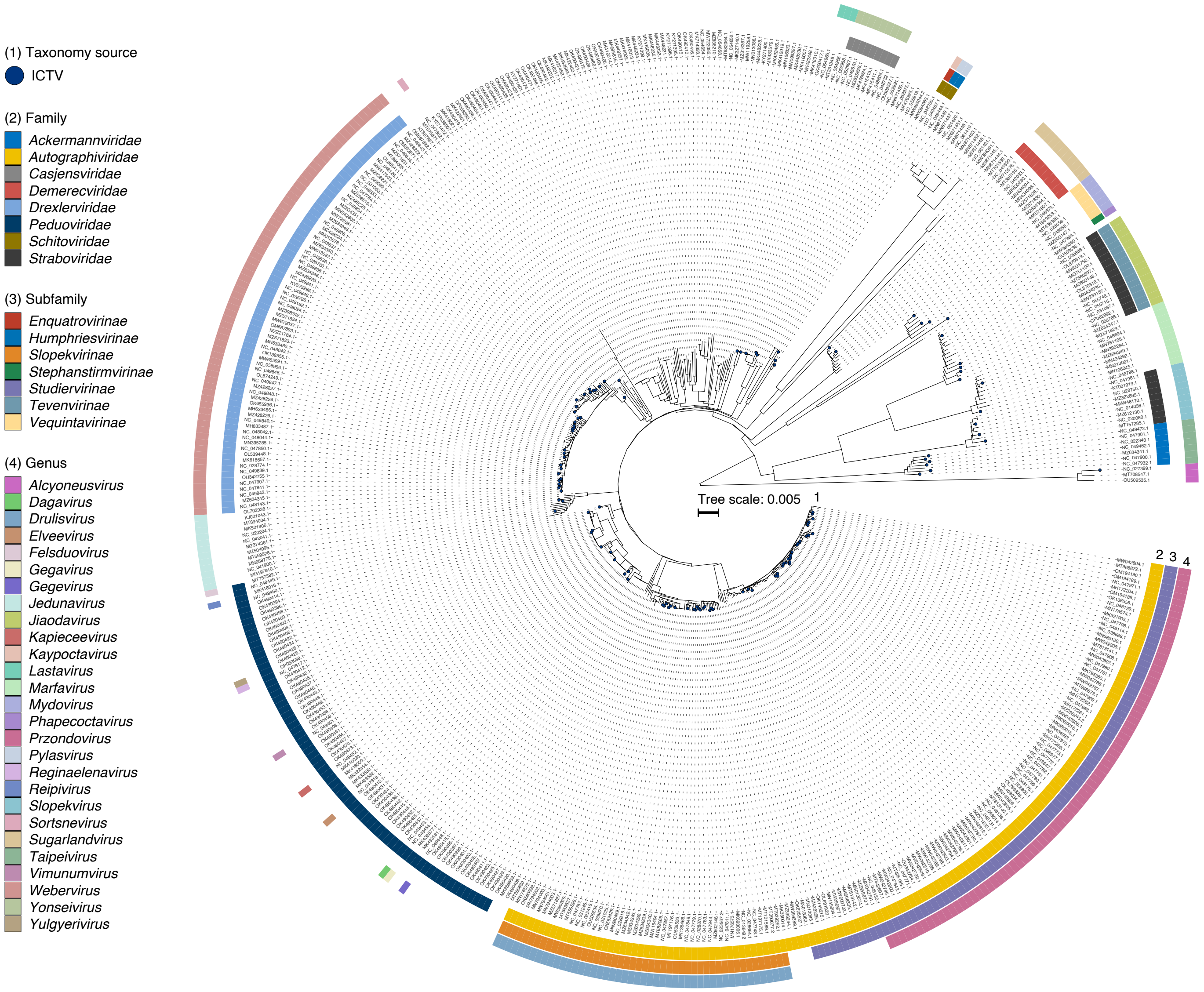


**Supplementary Figure S4**

Midpoint-rooted phylogenetic tree of $n$ = 480 *K. pneumoniae* phages based on pairwise distance matrix from gene presence/absence data.

The three external rings indicate the family, subfamily and genus rank, respectively. Genomes with available ICTV taxonomy are shown as dots on tip ends.
